# The effects of lipopolysaccharide-induced endotoxic shock on the intestinal microcirculatory perfusion: an experimental study in pigs

**DOI:** 10.1186/s40635-026-00891-8

**Published:** 2026-04-16

**Authors:** Moritz Flick, Brennan Schneider, Feras Hatib, Zhongping Jian, Bernd Saugel, Manuel Ignacio Monge García, Thomas W. L. Scheeren

**Affiliations:** 1 Department of Anesthesiology, Center of Anesthesiology and Intensive Care Medicine, University Medical Center Hamburg-Eppendorf, Hamburg, Germany; 2 Edwards Lifesciences, Irvine, CA USA; 3 Unidad de Cuidados Intensivos, Hospital Universitario Puerto Real, Puerto Real, Spain; 4 Department of Anesthesiology, University Medical Center Groningen, University of Groningen, Groningen, The Netherlands

**Keywords:** Sepsis, Tissue perfusion, Incident dark field illumination imaging, Critical care, Microcirculation, Microvascular flow index

## Abstract

**Background:**

Impairment of microcirculatory tissue perfusion is a central element of sepsis. Unfortunately, monitoring microcirculatory tissue perfusion to detect microcirculatory dysfunction at the bedside remains challenging. Therefore, the effects of sepsis and systemic infection on the intestinal microcirculatory tissue perfusion remain poorly understood.

**Methods:**

In this experimental study, lipopolysaccharide was infused in six healthy female adult Yorkshire cross-breed pigs to induce endotoxic shock. Subsequently, animals were primarily resuscitated using fluids and subsequently with additional norepinephrine. The intestinal microcirculation was monitored using videomicroscopy with incident dark field imaging and quantified using the microvascular flow index. We primarily analyzed changes of the intestinal microvascular flow index. Additionally, we investigated the relationship between the intestinal microvascular flow index and other hemodynamic variables using repeated measures correlation *r*_rm_(*n*).

**Results:**

The median (25th to 75th percentile) intestinal microvascular flow index decreased from 3.0 (2.9 to 3.0) at baseline to 2.5 (1.5 to 2.6) during endotoxic shock (*P* = 0.027). After resuscitation with fluids, the intestinal microvascular flow index increased to 2.8 (2.8–2.9) and after additional norepinephrine administration to 3.0 (2.9–3.0). Intestinal heterogeneity index increased from 0.1 (0 to 0.1) to 0.4 (0.4 to 0.5) during endotoxic shock and returned to baseline levels 0 (0 to 0.1) after resuscitation with fluids and norepinephrine. The correlation between the microvascular flow index and mean arterial pressure was *r*_rm_(32) = 0.39 (95% CI 0.054 to 0.64; *P* = 0.024), and between the microvascular flow index and cardiac *o*utput *r*_rm_(32) = 0.21 (95% CI − 0.133, 0.515; *P* = 0.223).

**Conclusions:**

In line with previous experimental studies, lipopolysaccharide-induced endotoxic shock impaired intestinal microcirculatory tissue perfusion and increased intestinal flow heterogeneity, both of which recovered after resuscitation with fluids and norepinephrine in this porcine model. Macrohemodynamic variables remained impaired after resuscitation and, therefore, correlated only weakly with the intestinal microvascular flow index. Thus, macrohemodynamic variables should not be used as a surrogate for microcirculatory tissue perfusion during endotoxic shock.

**Supplementary Information:**

The online version contains supplementary material available at 10.1186/s40635-026-00891-8.

## Introduction

Impairment of microcirculatory tissue perfusion is a central element of sepsis [1]. Impaired microcirculatory tissue perfusion is associated with organ injury and mortality in septic patients [2–4]. Unfortunately, monitoring microcirculatory tissue perfusion to detect microcirculatory dysfunction at the bedside remains challenging [5, 6].

Handheld videomicroscopy allows direct visualization and quantification of microcirculatory tissue perfusion [5]. Because of its accessibility, most studies performed in patients investigate the sublingual microcirculatory tissue perfusion [7, 8], which may not always reflect microcirculatory tissue perfusion of vital organs, e.g., the intestines [9]. The effects of sepsis and systemic infection on the intestinal microcirculatory tissue perfusion thus remain poorly understood.

Therefore, we performed this experimental animal study to investigate the effect of lipopolysaccharide (LPS)-induced endotoxic shock on the intestinal microcirculatory perfusion quantified by the microvascular flow index. Our hypothesis was that the microvascular flow index decreases during LPS-endotoxic shock compared to baseline. Additionally, we investigated the relationship between the intestinal microvascular flow index and other (macro)-hemodynamic variables.

## Materials and methods

### Study design

This experimental study was performed at the Edwards Research Center (Irvine, CA, USA) between June 2022 and December 2022. The study was approved by the United States Department of Agriculture (USDA; Certificate No. 93-R-0121) and the Institutional Animal Care and Use Committee at the Edwards Research Center. The study was performed in accordance with the USDA Animal Welfare Act regulations and the Guide for the Care and Use of Laboratory Animals (ILAR, NAP, Washington, DC, 2010, 8th edition).

The test facility is accredited by the Association for the Assessment and Accreditation of Laboratory Animal Care, International, and registered with the United States Department of Agriculture to conduct research with laboratory animals. We report the study according to the Animal Research: Reporting of In Vivo Experiments (ARRIVE) guidelines [10].

### Animal preparation and surgical procedures

We included eight healthy female adult Yorkshire cross-breed pigs aged between 3 and 4 months with a mean ± standard deviation (SD) body weight of 72.4 ± 1.6 kg. The pigs were maintained in temperature-controlled and humidity-controlled rooms with a typical light–dark cycle and given standard chow and tap water ad libitum. The animals were tested in sequential order. General anesthesia was induced with intramuscular administration of 4.4 mg/kg zolazepam, 2.2 mg/kg S-ketamine, and 1.1 mg/kg xylazine. After endotracheal intubation, the pigs were mechanically ventilated in volume-controlled mode targeting an end-tidal carbon dioxide between 35–40 mmHg. Initially, the animals were ventilated with an inspiratory percentage of 3.0 Vol% inhalational isoflurane and a mixture of oxygen (inspiratory fraction of 0.6–0.8) and air. The inhalational isoflurane was subsequently reduced to 1.5–2.5 Vol% to achieve hemodynamic stability. Routine monitoring with an electrocardiogram, peripheral oxygen saturation, and rectal temperature was applied; a urinary catheter was placed. An arterial catheter was inserted in the femoral artery and central venous catheters were placed in the femoral and internal jugular vein. A pulmonary artery catheter (Thermodilution Pace Port Catheter, Swan–Ganz; Edwards Lifesciences, Irvine, CA) was inserted into the pulmonary artery via the internal jugular vein guided by pressure waveform analysis.

All animals were given Ringer’s lactate solution at a rate of 2 ml/kg/h as baseline fluid. Body temperature was kept between 38 and 39 °C using a heating pad. Anesthesia depth and pain were assessed by vital sign monitoring and performing a jaw tone and toe pinch every 15 min for the duration of the experimentation. Depth of anesthesia was adjusted accordingly. Additional rocuronium was administered in case of spontaneous breathing activity.

### Study protocol

We performed a midline laparotomy and subsequently an enterotomy on the small intestine to insert the videomicroscope. All animals were stabilized with additional Ringer’s lactate and adjusted inhalational isoflurane concentration targeting a stroke volume variation between 10 and 20%, a mixed venous oxygen saturation (S_v_O_2_) > 60%, and a mean arterial pressure > 80 mmHg. We defined a stable baseline as heart rate and mean arterial pressure variation < 10% for at least 30 min.

Our protocol was based on established endotoxic shock models [11–14]. For inducing endotoxic shock, an initial priming was performed using LPS (E-Coli 055:B5, Sigma, St. Louis, MO, USA) with an infusion rate of 2 µg/kg/h that was increased to 4 µg/kg/h after 10 min until mean pulmonary artery pressure doubled from baseline. At this time, the LPS infusion was stopped for 30 min. Then, endotoxic shock was induced with LPS infusion with a starting rate of 6 µg/kg/h. After 10 min, the LPS infusion rate was increased to 12 µg/kg/h and after an additional 10 min to 24 µg/kg/h. We stopped the LPS infusion as soon as the mean arterial pressure decreased to < 50 mmHg or S_v_O_2_ to < 50%. “Shock” was defined as a mean arterial pressure ≤ 45 mmHg. To reverse shock, we subsequently administered a fluid bolus of 20 ml/kg Ringer’s lactate followed by a 10 ml/kg 6% Dextran infusion over 1 h. Following fluid resuscitation, norepinephrine infusion was started and titrated to return mean arterial pressure to the pigs’ individual baseline value.

### Microcirculation monitoring

Following enterotomy on the small intestine, we monitored the intestinal microcirculation at the level of the mucosa using videomicroscopy with incident dark field imaging (Cytocam; Braedius Medical, Huizen, the Netherlands) [15]. Video sequences were recorded together with macrohemodynamic variables at baseline, every thirty minutes until the animals were in shock, and then once after fluid resuscitation and after additional norepinephrine administration. At each time point, at least three 4-s video sequences were recorded. The video sequences were stabilized and subsequently reviewed for quality according to the microcirculation image quality score [16]. Specifically, we scored videos based on illumination, duration, focus, content, stability, and pressure with 0 points for optimal, 1 point for acceptable, and 10 points for unacceptable quality. Only videos with sufficient quality (total quality score < 10 points) were subsequently analyzed in random order by an investigator (MF) blinded to the animal and the time point. We assessed the microvascular flow index ranging from 0–3 (au; 0, no flow; 1, intermittent low; 2, sluggish flow; 3, normal flow) and the heterogeneity index [5]. The heterogeneity index was calculated as the highest minus the lowest microvascular flow index per quadrant divided by the mean microvascular flow index of the same video. We averaged the values from available video sequences from each animal at each time point for further analysis.

We also monitored regional tissue oximetry (S_t_O_2_) on the skin above the gracilis muscle by placing an adult near-infrared spectroscopy sensor (ForeSight sensor; Edwards Lifesciences, Irvine, CA, USA).

### Macrocirculation monitoring

The femoral arterial pressure waveform was recorded continuously using an arterial catheter (AcumenIQ sensor; Edwards Lifesciences, Irvine, CA, USA). Throughout the study, optimal damping of the arterial pressure waveform was carefully checked by intermittently fast flushing the line and checking the square wave test. Advanced macrohemodynamic variables including cardiac output and the Hypotension Prediction Index Software [17] were measured using pulse wave analysis (Hemosphere, Edwards Lifesciences).

Macrohemodynamic variables were recorded at baseline, every thirty minutes until the animals were in shock, and then once after fluid resuscitation and after additional norepinephrine administration. At each time point, arterial and venous blood gas analyses were performed, and SvO_2_ from the pulmonary artery catheter was recorded.

### Endpoints

The primary endpoint was the intestinal microvascular flow index during endotoxic shock. As secondary endpoints, we investigated the effects of endotoxic shock on macrohemodynamic variables (heart rate, blood pressure, cardiac output), and the association between the microvascular flow index and macrohemodynamic variables.

### Statistical analysis and sample size calculation

Categorical data are presented as absolute numbers (percentage), and continuous data are presented as median (25th, 75th percentile). We compared microvascular flow index measurements between time points with Wilcoxon signed-rank tests for paired measurements using MedCalc (MedCalc Software Ltd, Ostend, Belgium). The correlations between the microvascular flow index and other hemodynamic variables were assessed using repeated measures correlation (*r*_rm_(*n*)) [18, 19]. We expected a mean microvascular flow index of 2.9 with a standard deviation of 0.1 at baseline and a microvascular flow index of 2.0 (reflecting a change from ‘continuous’ to ‘sluggish’ capillary blood flow) with a standard deviation of 0.5 during the shock phase, resulting in an expected effect size of 1.96. Considering an alpha of 0.05 and a power of 0.9, a minimum sample size of 5 pigs was calculated. The sample size calculation was performed using G-Power (V3.1.9.7) [20].

## Results

**Fig. 1:**
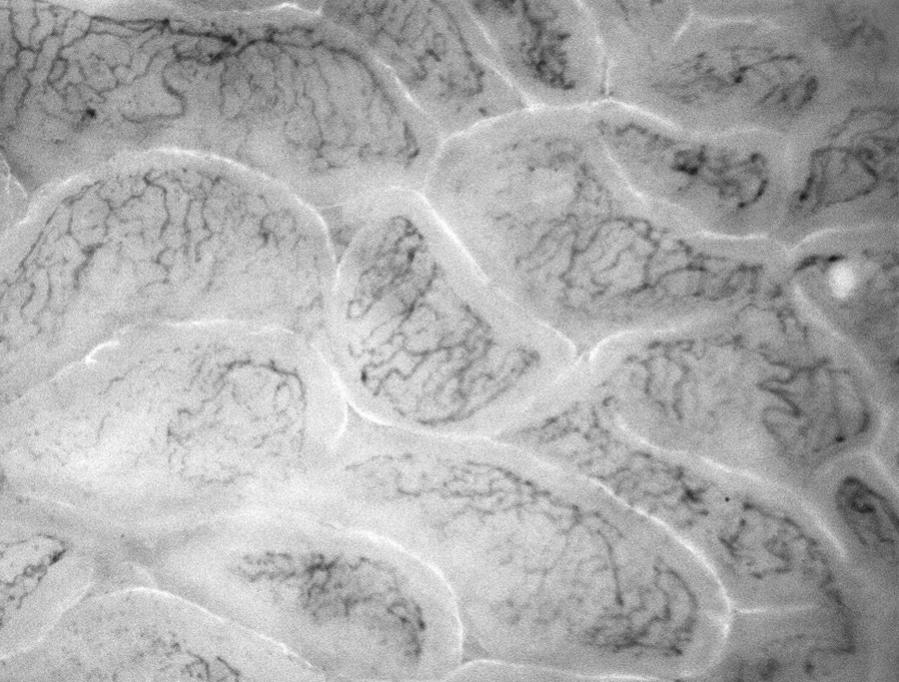
Image of the intestinal microcirculation

We included eight pigs but excluded two because no stable baseline was reached; thus, they were considered too unstable for the planned protocol. The study protocol was thus performed in 6 pigs, which were all included in the analysis. We recorded a total of 278 video sequences of the intestinal microcirculation, of which 219 (78%) were analyzed (Fig. 1).

At baseline, median (25th to 75th percentile) mean arterial pressure was 87 (81 to 90) mmHg, cardiac output was 7.7 (6.2 to 8.3) L min^−1^, and heart rate was 72 (64 to 75) min^−1^ (Table 1, Supplementary Fig. 1). In the shock phase, mean arterial pressure decreased to 46 (45 to 47) mmHg and cardiac output to 4.5 (4.3 to 4.6) L min^−1^, whereas heart rate increased to 95 (88 to 106) min^−1^ compared to baseline. After initial fluid resuscitation, mean arterial pressure was 50 (49 to 53) mmHg, cardiac output was 5.2 (4.7 to 5.4) L min^−1^, and heart rate was 106 (91 to 114) min^−1^. After additional administration of norepinephrine, mean arterial pressure increased to 83 (78 to 84) mmHg, cardiac output to 23.9 (20.8 to 25.7) L min^−1^, and heart rate to 143 (137 to 145) min^−1^.

**Table 1:** Hemodynamic measurements

	Baseline	30 min LPS infusion	60 min LPS infusion	90 min LPS infusion	Endotoxic shock	Fluid resuscitation	Fluid and norepinephrine resuscitation
Microvascular flow index, au	3.0 (2.9 to 3.0)	2.9 (2.8 to 2.9)	2.8 (2.7 to 2.9)	2.7 (2.3 to 2.8)	2.5^#^ (1.5 to 2.6)	2.8^†^ (2.8 to 2.9)	3.0 (2.9 to 3.0)
Heterogeneity index, au	0.1 (0 to 0.1)	0.1 (0.1 to 0.2)	0.2 (0.1 to 0.3)	0.2 (0.1 to 0.3)	0.4 (0.4 to 0.5)	0.2 (0.1 to 0.2)	0 (0 to 0.1)
Mean arterial pressure, mmHg	87 (81 to 90)	79 (74 to 85)	65 (64 to 67)	67 (63 to 69)	46 (45 to 46)	50 (49 to 53)	83 (78 to 84)
Heart rate, bpm	72 (64 to 75)	82 (75 to 85)	82 (70 to 89)	92 (79 to 98)	95 (88 to 106)	106 (91 to 114)	143 (137 to 145)
Cardiac output, L/min	7.7 (6.2 to 8.3)	7.1 (6.4 to 8.8)	8.0 (7.2 to 8.6)	8.0 (6.2 to 9.8)	4.5 (4.3 to 4.6)	5.2 (4.7 to 5.4)	23.9 (20.8 to 25.7)
Hypotension prediction index software, au	27 (19 to 45)	52 (30 to 74)	97 (96 to 98)	95 (92 to 98)	100 (100 to 100)	100 (100 to 100)	56 (53 to 68)
Lactate, mmol/L	1.9 (1.9 to 2.0)	1.9 (1.8 to 2.1)	1.7 (1.6 to 2.2)	2.3 (1.8 to 2.8)	2.6 (2.5 to 2.8)	4.0 (3.8 to 4.3)	4.1 (3.7 to 5.4)
Regional tissue oxygen saturation, %	60 (51 to 63)	55 (54 to 59)	52 (52 to 58)	55 (53 to 59)	56 (51 to 60)	69 (62 to 75)	60 (55 to 63)
Mixed venous oxygen saturation, %	73 (70 to 75)	75 (73 to 79)	79 (77 to 81)	80 (78 to 82)	77 (73 to 78)	77 (73 to 79)	84 (84 to 85)
Norepinephrine dose*, µg/kg/min	–	–	–	–	–	–	0.75 (0.67 to 0.85)

Values are presented as median (25th to 75th percentile)

*LPS: lipopolysaccharide*

^*^mean norepinephrine dose during resuscitation

^*#*^*indicates a P-value* < *0.05 compared to baseline*

*†indicates a P-value* < *0.05 compared to previous measurement point*

**Fig. 2:**
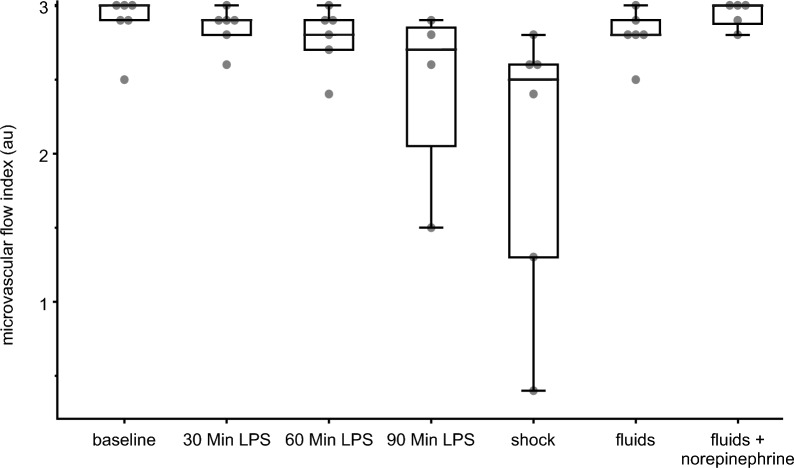
Box plots of the intestinal microvascular flow index

All animals had normal intestinal microcirculatory tissue perfusion at baseline with a median microcirculatory flow index of 3.0 (2.9 to 3.0) and heterogeneity index of 0.1 (0 to 0.1) (Table 1, Fig. 2, Supplementary Fig. 1). During endotoxic shock, the intestinal microvascular flow index decreased to 2.5 (1.5 to 2.6) (*P* = 0.027 compared to baseline) and the heterogeneity index increased to 0.4 (0.4 to 0.5). Two of the six animals showed signs of a severe impairment of the intestinal microcirculatory tissue perfusion with microvascular flow indices of 0.4 and 1.3. After resuscitation with fluids, the intestinal microvascular flow index increased to 2.8 (2.8–2.9), and the heterogeneity index decreased to 0.2 (0.1 to 0.2). After additional norepinephrine administration, the microvascular flow index was 3.0 (2.9–3.0) and the heterogeneity index was 0 (0 to 0.1) –values similar to those at baseline.

There was an increase in lactate from 1.9 (1.9 to 2.0) mmol/L at baseline to 4.1 (3.7 to 5.4) mmol/L after fluid and norepinephrine resuscitation. There was no clear trend in regional tissue oxygenation or central venous oxygen saturation (Table 1, Supplementary Fig. 1).

The correlation between the microvascular flow index and mean arterial pressure was *r*_rm_(32) = 0.39 (95%CI 0.054 to 0.64; *P* = 0.024), and between the microvascular flow index and cardiac output *r*_rm_(32) = 0.21 (95%CI − 0.133, 0.515; *P* = 0.223). There was no clinically meaningful correlation between the microvascular flow index and other hemodynamic variables (Table 2).

**Table 2:** The correlation between the microvascular flow index and other hemodynamic variables

	Repeated measures correlation	95% confidence interval	*P*-value
Heterogeneity index, au	− 0.81	− 0.9 to − 0.646	< 0.001
Mean arterial pressure, mmHg	0.39	0.054 to 0.64	0.024
Heart rate, bpm	0.12	− 0.227 to 0.441	0.496
Cardiac output, L/min	0.21	− 0.133 to 0.515	0.223
Regional tissue oxygen saturation, %	0.1	− 0.243 to 0.427	0.559
Mixed venous oxygen saturation, %	− 0.13	− 0.452 to 0.213	0.447
Lactate, mmol/L	0.20	− 0.153 to 0.509	0.263

## Discussion

In this experimental study, LPS-induced endotoxic shock reduced microcirculatory tissue perfusion and increased microvascular flow heterogeneity. Initial resuscitation with fluids recovered intestinal microcirculatory tissue perfusion, but not macrohemodynamic variables. Therefore, the correlation between the microvascular flow index and mean arterial pressure and the microvascular flow index and cardiac output was weak.

Our present findings are fully concordant with several previous studies investigating intestinal microcirculatory tissue perfusion in experimental endotoxic shock [21–24]. A study with 7 sheep investigating the microvascular flow index in the intestinal mucosa, the intestinal serosa, and sublingually during LPS-induced endotoxic shock found substantial decreases in all measurement sites during endotoxic shock [21]. Interestingly, microcirculatory tissue perfusion in the intestinal serosa and sublingually recovered after resuscitation, but not that in the intestinal mucosa. Similarly, a trial with 20 sheep randomized to resuscitation with or without nitroglycerin infusion also found impaired intestinal microcirculatory tissue perfusion during endotoxic shock [22]. Another study from the same group investigating LPS-induced shock in 12 sheep showed predominantly renal microcirculatory impairment with less distinct changes in the intestinal villi [23]. Similar results were also shown in rats [24]. Our study does not demonstrate unexpected or contradictory findings, but rather confirms these previous findings. The results of our present study contribute to the existing body of evidence regarding intestinal microcirculatory tissue perfusion impairment during systemic inflammation. However, in both sheep studies, the intestinal microcirculation did not fully recover after resuscitation. This contrasts with our study, as we observed a high intestinal microvascular flow index and low heterogeneity—with similar values to those observed at baseline—after resuscitation. These observed differences may—in part—be explained by the fact that the previous studies waited an hour prior to starting resuscitation. Even though the intestinal microvascular flow index at baseline was high in all our animals, we observed a high variability in microvascular flow index values during the endotoxic shock phase—which was previously reported in other experimental studies, but is also commonly observed in clinical studies [7, 25]. A potential explanation for the observed high variability in intestinal microvascular flow index during LPS-induced endotoxic shock may be that some animals better compensate than others. Another potential reason for the observed variability in the intestinal microvascular flow index may also be observer dependence of handheld videomicroscopy.

As expected, LPS-induced endotoxic shock not only impaired microcirculatory tissue perfusion, but also the macrocirculation—reflected by a drop in mean arterial pressure and cardiac output with a simultaneous increase in heart rate. Initial resuscitation with fluids did not completely reverse hypotension. Thus, additional norepinephrine infusion was necessary to stabilize blood pressure in all cases but also resulted in a hyperdynamic circulation with tachycardia and very high cardiac output. The overall correlation between intestinal microcirculatory flow index and macrohemodynamic variables was, therefore, weak in our study. Nonetheless, the correlation between microcirculatory tissue perfusion and the macrocirculation remains a matter of ongoing debate [26, 27]. Some experimental as well as clinical studies have suggested a stronger correlation between microcirculatory tissue perfusion variables and macrohemodynamic variables [28, 29], whereas others have not [9, 30, 31]. These conflicting findings may be in part explained by differences in the assessment of microcirculatory tissue perfusion, e.g., intestinal versus sublingual [9]. Interestingly, in our study, regional oxygen saturation and mixed venous oxygen saturation, which are sometimes considered as surrogates for microcirculatory tissue perfusion, also did not correlate with the intestinal microvascular flow index. This underlines the importance of direct monitoring of microcirculatory tissue perfusion—and not the use of surrogate variables.

This was an experimental study with LPS-induced endotoxic shock in otherwise healthy pigs and its results naturally cannot be directly applied to human patients. In this model, we started resuscitation with fluids only and later added norepinephrine as a vasopressor. We, therefore, could not describe the effects of norepinephrine independently of that of fluid administration during resuscitation. However, our results indicate that high norepinephrine dosages did not harm the intestinal microcirculation.

Naturally, the results are not directly transferable to a clinical setting with multi-morbid septic shock patients. We monitored intestinal microcirculatory perfusion using incident dark field imaging to visualize the intestinal microcirculation. In contrast to previous generations of handheld videomicroscopes, incident dark field imaging provides a larger field of view and overall better image quality [15]. Even though video sequences were recorded with great care and analyzed by a blinded experienced investigator, there is a risk of observer bias when using videomicroscopy to assess microcirculatory tissue perfusion. To quantify intestinal microcirculatory perfusion, we analyzed the microvascular flow index and the heterogeneity index. In studies investigating the sublingual microvascular flow index, absolute values < 2.6 have been associated with higher mortality in critically ill [32]. For the heterogeneity index, values of 0.5—that we also observed in our study—have been previously reported in critically ill patients with prolonged capillary refill time [33]. Subjective vessel detection and grading of flow—instead of automated objective analysis—reduces the interpretability. Further, the use of additional methods to assess the microcirculation would have allowed a more comprehensive description of the microcirculatory alterations.

Overall, this study contributes important information to our understanding of microcirculatory tissue perfusion during severe systemic inflammation. Monitoring intestinal microcirculatory perfusion is rarely feasible in clinical practice and our current findings underline the importance of microcirculatory impairment during systemic inflammation. Additionally, our results underline previous findings that systemic hemodynamic variables cannot be used as a surrogate for microcirculatory tissue perfusion and the necessity for a direct assessment.

In line with previous experimental studies, LPS-induced endotoxic shock impaired intestinal microcirculatory tissue perfusion and increased flow heterogeneity, which recovered after resuscitation with fluids and norepinephrine in this porcine model. Macrohemodynamic variables remained impaired after resuscitation and, therefore, correlated only weakly with the intestinal microvascular flow index. Thus, macrohemodynamic variables should not be used as a surrogate for microcirculatory tissue perfusion during endotoxic shock.

## Supplementary Information

Additional file1 (PDF 265 KB)

## Data Availability

The data set analyzed in this study is available from the corresponding author upon reasonable request.
