# Supplementary figures and images for: The effects of lipopolysaccharide-induced endotoxic shock on the intestinal microcirculatory perfusion: an experimental study in pigs
Source: Intensive Care Med Exp. 2026 Apr 16;14:47. doi: 10.1186/s40635-026-00891-8 (PMC13087001; doi:10.1186/s40635-026-00891-8)

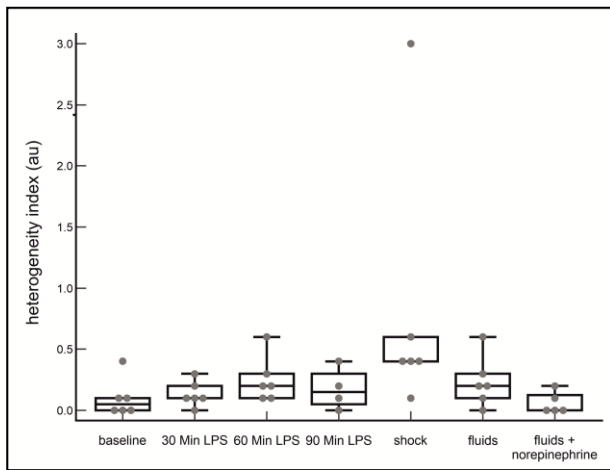

a)

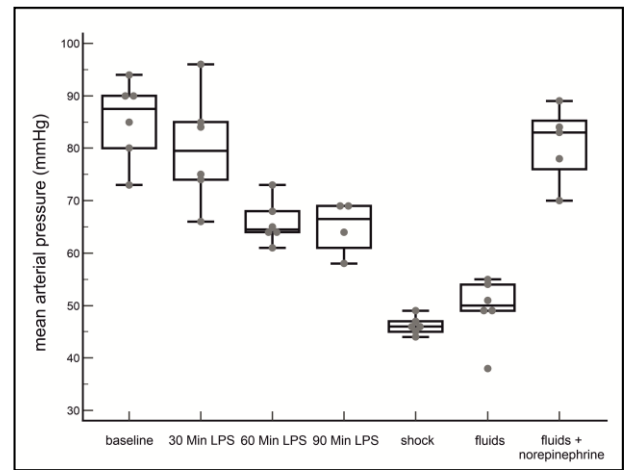

b)

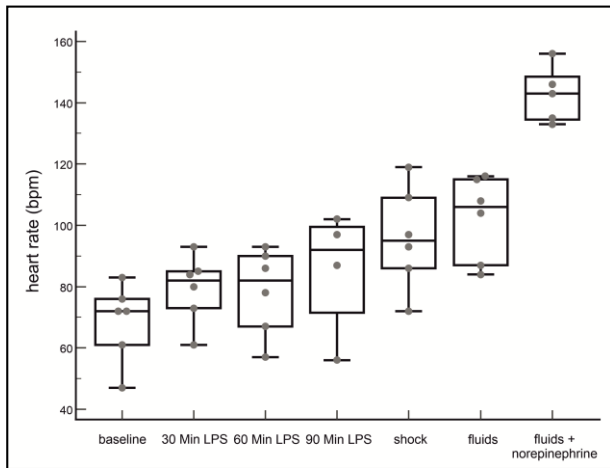

c)

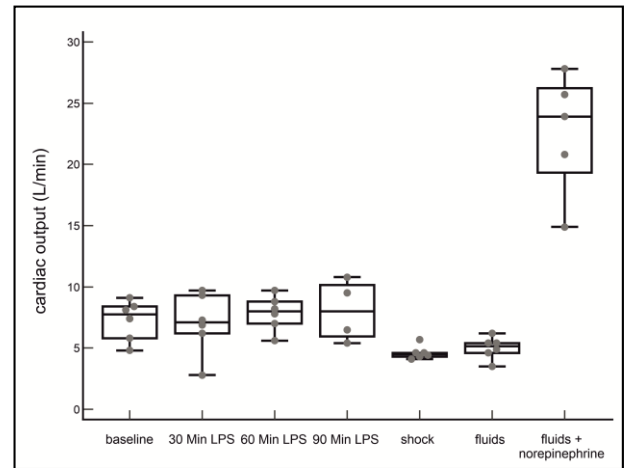

d)

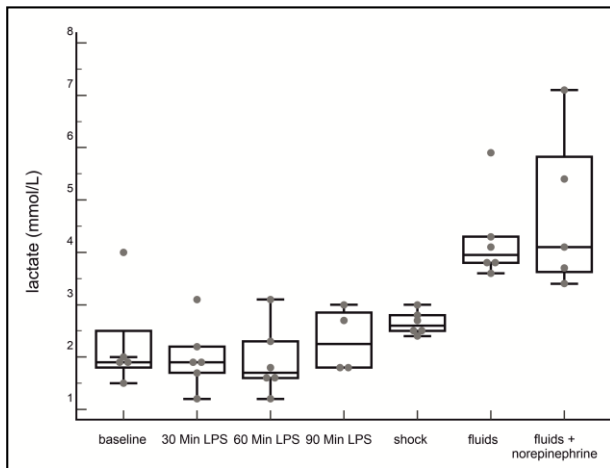

e)

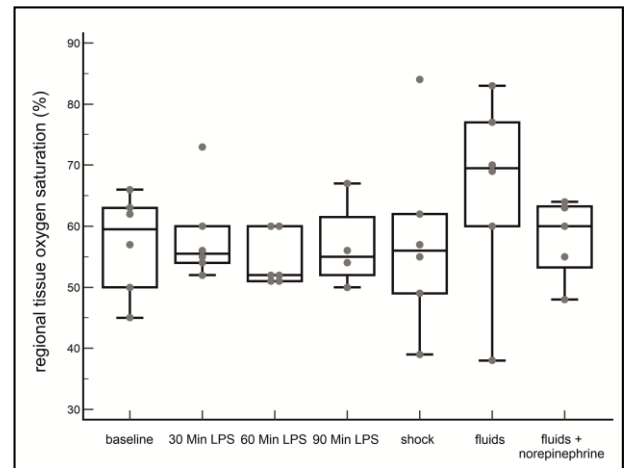

f)

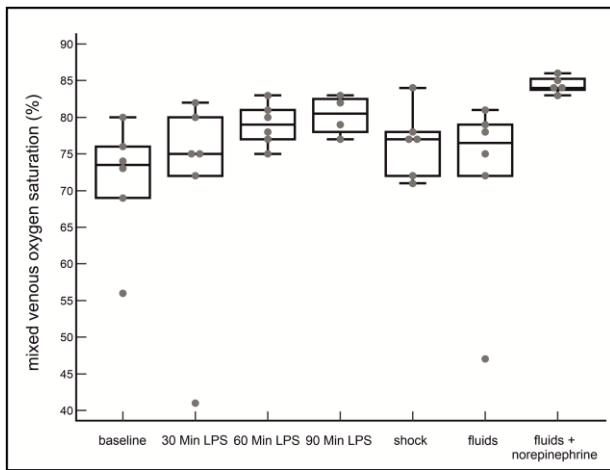

g)

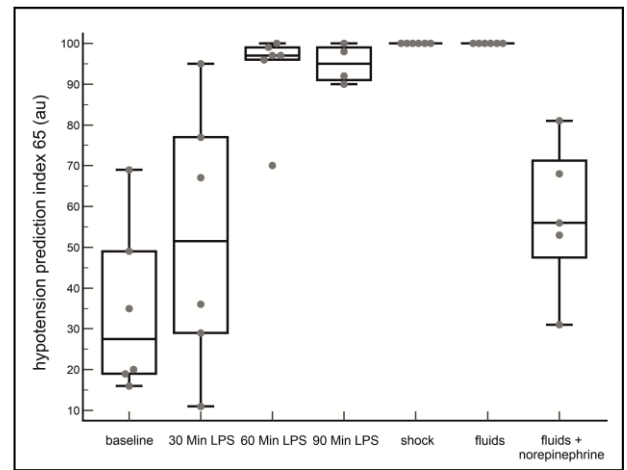

h)

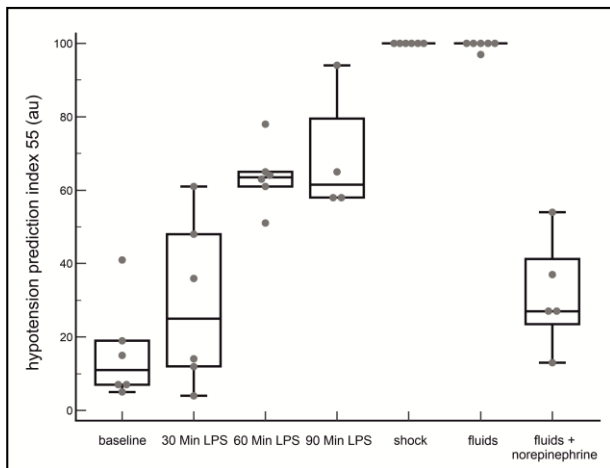

i)

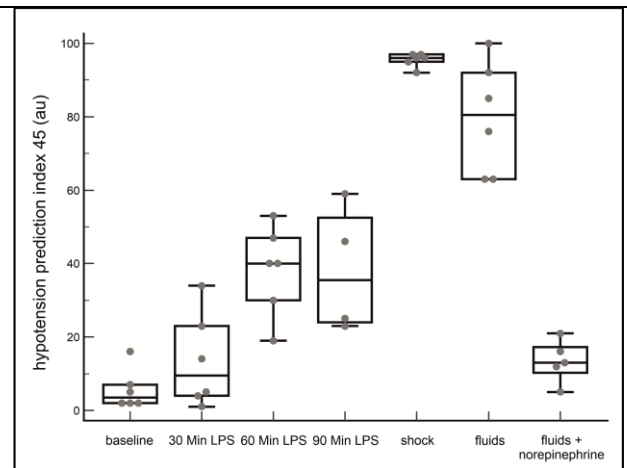

j)

**Supplementary Figure1 Box plots of additional hemodynamic variables**

Supplement: Supplementary file 1 — Additional file1 (PDF 265 KB) [file 40635_2026_891_MOESM1_ESM.pdf]
